# Prevalence and Associated Factors of Visual Hallucinations in Patients with Vascular Cognitive Impairment

**DOI:** 10.1155/2021/8866763

**Published:** 2021-01-09

**Authors:** Chih-Lin Chen, Min-Hsien Hsu, Chao-Hsien Hung, Pai-Yi Chiu, Chung-Hsiang Liu

**Affiliations:** ^1^Department of Neurology, Chang Bing Show Chwan Memorial Hospital, Changhua 505, Taiwan; ^2^Department of Neurology, Show Chwan Memorial Hospital, Changhua 500, Taiwan; ^3^Department of Nursing, College of Nursing and Health Sciences, Da-Yeh University, Dacun, Changhua 515, Taiwan; ^4^Department of Neurology, China Medical University Hospital, Taichung 404, Taiwan

## Abstract

Visual hallucinations (VHs) are striking features for dementia, especially dementia with Lewy bodies (DLB). We aimed to study the frequency and associated factors of VH in vascular cognitive impairment (VCI) and investigate the feasibility of clinically diagnosing the mixed pathology of VCI with DLB. This is a multicentre registration study. A consecutive series of VCI patients with and without dementia were enrolled. Frequency of VH and associated factors, including age, gender, education, disease severity, DLB clinical features, vascular risk factors, cognitive function, and neuropsychiatric symptoms, were compared between VCI with VH (VH+) and without VH (VH−). Among the 1281 patients analysed, 155 (12.1%) had VH. The VH+ group was older (*t* = 5.07; *p* < 0.001), was more likely to be female (*χ*^2^ = 13.46; *p* < 0.001), and has a higher clinical dementia rating (*χ*^2^ = 70.51; *p* < 0.001). After adjusting for age, gender, and disease severity, the VH+ group had poorer cognition and more severe neuropsychiatric symptoms. The VH+ group was more associated with DLB features in fluctuating cognition (OR = 2.48; *p* < 0.001), parkinsonism (OR = 1.85; *p* = 0.001), rapid eye movement (REM) behavioral disorder (OR = 4.56; *p* < 0.001), and ≧2 DLB core features (OR = 26.01; *p* < 0.001). VCI patients with VH tend to have more severe dementia, neuropsychiatric symptoms, and poorer cognitive function. Additionally, highly associated with clinical DLB features in VCI with VH raised the possibility of mixed pathology with DLB in this group. More than two core features in VCI might help in diagnosing a mixed pathology with DLB.

## 1. Introduction

Vascular cognitive impairment (VCI) or vascular dementia (VaD) is the second most common form of dementia [1]. Visual hallucinations (VHs) occasionally occur in patients with VCI, having a great impact on both patients and their caregivers [2–4]. Robust evidence has shown that VH is more common in patients that have dementia with Lewy bodies (DLB) than in Alzheimer's disease (AD), VaD, or other forms of dementia [2, 5–7]. Therefore, VH, along with fluctuations in cognition, parkinsonism, and rapid eye movement (REM) sleep behavior disorder (RBD), is the core clinical feature for clinically diagnosing DLB. Previous studies have shown that prevalence of characteristic VH (complex, well-formed, and detailed VH) is much higher in DLB compared to other forms of dementia [5–8]. In our previous research article, we also provided evidence of VH in AD should also be considered the mixed pathologies with DLB [8]. However, few studies have addressed the prevalence and association factors of VH in patients with VCI or other non-DLB dementia [2].

The connection between the core clinical features for diagnosing DLB with Lewy body pathology is based mainly on the findings of clinical and pathological studies of DLB. These demonstrated relatively good specificity, accuracy, and variable sensitivity [9–11]. This is revised from the previous consensus criteria, which excluded DLB in subjects with CVD or other brain disorders [12, 13]. The newest consensus criteria for DLB in 2017 stated that patients with DLB and comorbidities such as CVD or other brain disorders should not be excluded from potentially having DLB and that a mixed pathology should be considered [7]. Therefore, it is reasonable to propose in cases with VCI that if there is one typical core feature for diagnosing DLB, i.e., VH, mixed pathology with DLB should be investigated if at least one of the other core clinical DLB features or indicative biomarkers is present. According to the consensus criteria for DLB, two core features or one core feature plus one indicative biomarker is required for diagnosing probable DLB [7]. Furthermore, clinical-pathological studies on non-DLB dementia also provide evidence for mixed pathology of AD with DLB in cases where AD is comorbid with core DLB features [8, 14–16].

Diagnosing VH in dementia, especially in an amnesia-predominant disease like AD, is often confounded with a false memory of remote events due to impairment of source monitoring [17–19]. In that case, VH is usually less complex not persistent and is associated with delusions [20, 21]. This makes it difficult to differentiate with a remote memory of confabulations. However, complex, well-formed, and detailed VH, which is considered more characteristic for VH in DLB, should be more specific than actual VH. This type of impairment could be considered as a target feature for predicting mixed Lewy body dementia and other brain disorders.

To investigate the possible mixed Lewy body pathology in VCI with VH, in this study, only characteristic complex, well-formed, and detailed VHs were regarded as DLB type VH. These patients were allocated to the VH+ group. Due to the high specificity of clinically diagnosing DLB according to the consensus criteria, we proposed that by observing its association with other DLB clinical features, we can predict the possible mixed Lewy body pathology in VCI with VH. Besides, we also investigated the associated factors of VH in VCI including the demographical data, VCI subtypes, CVD subtypes, cognitive, neuropsychiatric, and vascular risk factors. Additionally, we investigated possible explanations of the associations.

## 2. Materials and Methods

### 2.1. Participants

This is a cross-sectional retrospective study. From October 2015 to July 2019, a consecutive series of participants from 3 regional hospitals in Taiwan were registered. A diagnosis of VCI was made according to the criteria for probable VaD, possible VaD, and vascular mild cognitive impairment (MCI) in the 2011 American Heart Association/American Stroke Association (AHA/ASA) criteria for VCI [22]. All patients had received at least a cerebral computed tomography (CT) or a cerebral magnetic resonance imaging (MRI) and a set of blood screening tests for ruling out other possibilities of cognitive decline. The following information was used in this study:

1. Age, gender, education, disease duration, vascular risk factors (VRFs), and current medication
2. Dementia severity using the Clinical Dementia Rating (CDR) scale and the sum of boxes of CDR (CDR-SB) [23]
3. Clinical Lewy body dementia (LBD) features, including cognitive fluctuations, parkinsonism, RBD, auditory hallucinations (AHs), delusions, and depression [7]
4. Cognitive performance on the Montreal Cognitive Assessment (MoCA) [24] and the Cognitive Abilities Screening Instrument, Chinese version (CASI C-2.0) [25]
5. Activities of daily living assessment by Instrumental Activities of Daily Living (IADL) scale [26]
6. Neuropsychiatric symptoms in the 12-item version of the Neuropsychiatric Inventory (NPI) based on observations within the past month [27]

### 2.2. Assessment of VH and Other DLB Core Clinical Features

Each patient and his or her primary caregiver were interviewed by a trained neuropsychologist for assessing VH and other core clinical features. VH was diagnosed when a clinical history of recurrent well-formed, complex, and detailed VH was present. The fluctuation of cognition was diagnosed when a Mayo Fluctuation Composite Score (MFCS) > 2 was present [28]. Parkinsonism was diagnosed when bradykinesia plus at least one of the following was present: resting tremor, rigidity, and instability. RBD was diagnosed according to the minimal criteria for clinically diagnosing RBD [29].

### 2.3. Data Analysis

For the statistical analyses, SPSS version 22.0 for Windows (IBM, SPSS Inc., Chicago) was used. Comparisons were made between the VH+ and VH- groups in terms of the demographic data, including CDR, clinical features, IADL, MoCA, CASI, and composite scores of the NPI. All data were analysed using an independent *t*-test, and the odds ratios (ORs) were adjusted for age, gender, and dementia severity. VCI subtypes (vascular MCI, probable VaD, and possible VaD) and CVD subtypes (multi-infarct, strategic infarct, subcortical lacunes, Binswanger disease, complex combination, hemorrhage, and others) were analysed using the chi-square test. DLB core clinical features (fluctuation, parkinsonism, and RBD), VRFs, and current medication were analysed using the chi-square test. All ORs were adjusted for age, gender, and dementia severity. To compare the associations between clinical symptoms/sign characteristic of DLB and neuroimaging variables between the VH+ and VH- groups, all ORs were adjusted for age, gender, and dementia severity.

### 2.4. Ethical Considerations

The participants were selected from a dementia database of Show Chwan Healthcare System. The study design was retrospective, and the data were analysed anonymously. The Committee for Medical Research Ethics of Show Chwan Memorial Hospital reviewed the project, and the Data Inspectorate approved the study.

## 3. Results

Among the 1281 patients with VCI, 155 (12.1%) had VH and 1126 (87.9%) did not. Compared with the VH- group, the VH+ group was older (79.6 vs. 75.2; *t* = 5.07 and *p* < 0.001), more likely to be female (63.2% vs. 47.9%; *χ*^2^ = 13.46 and *p* < 0.001), and had a higher dementia severity according to the CDR (*χ*^2^ = 70.51 and *p* < 0.001). After adjusting for age, gender, and dementia severity by CDR, poorer IADL (OR = 0.85; *p* = 0.002), higher prevalence of dementia (OR = 4.43; *p* < 0.001), CDR-SB (OR = 9.67; *p* < 0.001), CASI (OR = 1.02; *p* = 0.012), and total score of NPI (OR = 1.06; *p* < 0.001) were found in the VH+ group compared to the VH- group. Disease duration, VCI subtypes, and CVD subtypes were not different between the VH+ and VH- groups. Comparisons between the demographic and background data are summarized in Table 1.

Table 2 showed the clinical manifestations of VCI patients with or without VH in terms of DLB features, current medications, and VRFs adjusted for age, gender, and disease severity by CDR. Compared with the VH- group, the VH+ group had a significantly higher prevalence of all LBD features including cognitive fluctuations (OR = 2.48; *p* < 0.001), parkinsonism (OR = 1.85; *p* = 0.001), RBD (OR = 4.56; *p* < 0.001), and ≧2 DLB core features (OR = 26.01; *p* < 0.001). Compared with the VH- group on current medication, the VH+ group was significantly more likely to be on anti-Parkinson's medication (OR = 2.35; *p* = 0.010). Compared with the VH- group on VRFs, the VH+ group was significantly more likely to have hypertension (OR = 1.47; *p* = 0.032), diabetes (OR = 1.86; *p* = 0.001), and heart disease (OR = 2.12; *p* = 0.002).

Table 3 demonstrates the relationship between the cerebral CT/MRI of the VH+ group and the VH- group. After adjusting for age, gender, and dementia severity by CDR, the VH+ group had a significantly higher MTA scale (OR = 1.27; *p* = 0.010). The Fazekas scale, global atrophy scale, cerebral microbleeds (cortical, subcortical, or total), and Evans' index were not different between the two groups.

**Figure 1:**
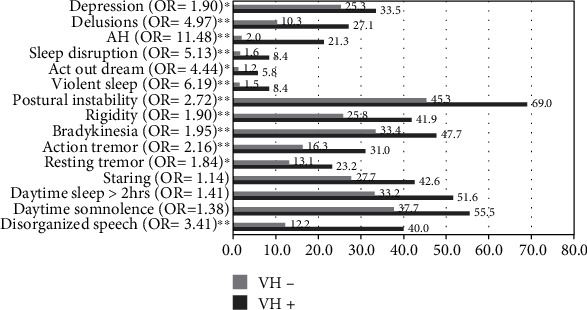
Percentage frequency of clinical feature characteristic of Lewy body dementia of VH+ compared to VH−. AH: auditory hallucination; ORs of variables were adjusted for age and disease severity as per the Clinical Dementia Rating scale. ^∗^*p* < 0.01; ^∗∗^*p* < 0.001.

Figure 1 demonstrates the percentage frequency of clinical symptom/signs that are characteristic of the DLB of VH+ compared to VH−. After having adjusted for age, gender, and disease severity, the VH+ group had a higher frequency in almost all the features including depression (OR = 1.90; *p* < 0.01), delusion (OR = 4.97; *p* < 0.001), auditory hallucinations (OR = 11.48; *p* < 0.001), sleep disruption (OR = 2.70; *p* < 0.001), acting out in dreams (OR = 4.44; *p* < 0.01), violent sleep (OR = 6.19; *p* < 0.001), postural instability (OR = 2.72; *p* < 0.001), rigidity (OR = 1.90; *p* < 0.001), bradykinesia (OR = 1.95; *p* < 0.001), action tremor (OR = 2.16; *p* < 0.001), resting tremor (OR = 1.84; *p* < 0.01), and disorganized speech (OR = 3.41; *p* < 0.001).

## 4. Discussion

Mixed degenerative pathologies, for example, Alzheimer's or Lewy body disease with cerebrovascular disease are highly prevalent according to pathological studies [30–33]. Some of these studies also revealed a high prevalence of cerebrovascular pathologies in the brain of patients with LBD [31–33]. Likewise, Lewy body pathology has been found in VaD autopsy cases [34]. However, most of the mixed pathological dementia cases were diagnosed postmortem. How to make a clinical diagnosis while alive is becoming an important issue. For studying nonmotor symptoms associated with vascular parkinsonism, Levin et al. proposed that nonmotor symptoms such as hallucinations may indicate another diagnosis or mixed pathology [35]. Our findings are consistent with this. In this study, at least two core features for clinically diagnosing DLB were much higher in the VCI with the VH group compared to the non-VH group with an OR 26. All of the core features (fluctuations, RBD, and parkinsonism) were much higher in the VH group. Therefore, by highlighting the diagnostic differences between typical DLB type VHs (complex, well-formed, and detailed VH), we have provided a way for clinically diagnosing mixed DLB in other brain disorders such as VCI. We consider these findings as to the most important of the current study.

Besides this, our study focused on the associated factors of VH in VCI. We have had several other significant findings. Firstly, typical DLB type VH is not frequently observed in patients with VCI, with a prevalence of only 12.1%. VH in VCI was found to be more prevalent in those that are older, female, and have advanced dementia. These findings are consistent with our previous study that examined the gender differences of VH with DLB [36] as well as several other studies that focused on psychotic symptoms in vascular or degenerative dementia [4, 15, 37]. Even if these demographical factors have been adjusted for, the VH+ group still presented poorer cognitive function, ADL, and more severe neuropsychiatric symptoms. This may impact the quality of life of both patients and caregivers and also result in a greater caregiver burden [38, 39]. For the diagnosis of probable DLB, at least 2 core clinical features or one core clinical feature plus at least one indicative biomarker is necessary. Therefore, we analysed the symptoms of the associated core features (parkinsonism, fluctuation, and RBD) demonstrated on Figure 1 and found that symptoms of RBD have highest odds ratio (OR); however, the prevalence of RBD in VCI is relatively low (less than 5%) which might offset the diagnostic power of RBD in mixed DLB with VCI. On the contrary, although ORs for association of parkinsonism or fluctuation are both somewhat lower than RBD in VCI with VH, the prevalence of most of the symptoms of parkinsonism or fluctuation is higher than 50% which raised the power of the diagnosis of mixed pathologies of DLB in VCI with VH. Secondly, a significantly higher prevalence of VRFs, including hypertension, diabetes, and heart disease, was associated with the VH+ group in this study. However, hyperlipidemia was not. These results can be considered novel findings for VH in VCI. Similar studies on VRF association with neuropsychiatric symptoms in MCI or dementia have seldom been studied and remain controversial. Besides, none of them directly addressed to VH in VCI. A previous study concluded that VRFs were important modifiers of the risk of psychosis in AD [40]. Another study revealed that cholesterol is a significant factor for NPS occurring in AD [41]. A study from Cache County found that hypertension was associated with a higher risk of delusions, anxiety, and agitation/aggression, but not psychotic symptoms in AD. No significant associations were observed between neuropsychiatric symptoms and diabetes, hyperlipidemia, or heart disease [42]. Hypertension and previous CVD were the most prevalent risk factors for neuropsychiatric symptoms in VaD and mixed-type dementia [43]. Thirdly, it was revealed in this study that only medial temporal lobe atrophy had a modest association with VH in VCI. White matter lesions (WMLs) according to the Fazekas scale, global atrophy, cerebral microbleeds, and ventricle sizes were not associated. These findings were not consistent with previous studies regarding degenerative dementia that showed that WHLs are highly associated with neuropsychiatric symptoms in AD, DLB, or Parkinson disease with dementia (PDD) [44–46].

This study has several limitations. Firstly, this is a cross-sectional association study. Therefore, causal relationships to VH in VCI cannot be made. Secondly, dopamine transporter imaging, I123MIBG, or polysomnography, being indicative biomarkers for DLB, were not done in this study. The findings of the study were analysed purely according to the clinical manifestation plus brain structure imaging. Therefore, objective evidence of the mixed pathology with LBD was lacking. Thirdly, no pathological data was able to prove the diagnostic accuracy of clinical diagnosing mixed Lewy body pathology in VCI with VH.

## 5. Conclusions

In conclusion, VCI patients with VH tend to have more severe dementia, neuropsychiatric symptoms, and poorer cognitive function. Besides, there is a possibility of mixed pathology with DLB in this group due to the clinical DLB features in VCI with VH being associated. More than two core features in VCI might help for diagnosing mixed pathology with DLB. VH in VaD or VCI has not been widely studied in the literature. However, the underlying pathophysiology of VH, especially associated with typical DLB type VH (complex, well-formed, and detailed) in VCI, could be due to a mixed pathology with Lewy body disease such as DLB. Hence, we propose this novel concept along with a method for diagnosing mixed VCI with DLB. Further biomarker and pathological studies are warranted to prove its feasibility.

## Figures and Tables

**Table 1:** Demographic and background characteristics of VCI patients with or without visual hallucinations (VHs) adjusted for age, gender, and disease severity by CDR.

	Mean (SD, range)		Nonadjusted		Adjusted	
	VH+, mean (SD)	VH−, mean (SD)	*t*/*χ*^2^	*p*	OR (95% CI)	*p*
*N*	155	1126				
Age, years	79.6 (8.9)	75.2 (10.3)	5.07	<0.001	NA	
CDR 0.5/1/2/3	12/40/54/49	407/311/256/152	70.51	<0.001	NA	
Gender, female (%)	98 (63.2)	535 (47.9%)	13.46	<0.001	NA	
Education, years	3.4 (3.9)	5.0 (4.5)	-4.41	<0.001	0.97 (0.93-1.02)	NS
Duration, years	3.9 (6.0)	3.1 (5.3)	1.82	NS	1.02 (0.99-1.05)	NS
Dementia, *N* (%)	152 (98.1)	939 (83.4)	23.22	<0.001	4.43 (1.35-14.53)	<0.001
CDR-SB	11.6 (4.8)	7.6 (5.5)	9.67	<0.001	1.16 (1.04-1.28)	0.007
IADL	1.0 (1.9)	2.9 (3.1)	-10.65	<0.001	0.85 (0.76-0.94)	0.002
MoCA	5.1 (5.5)	8.5 (7.3)	-6.86	<0.001	1.02 (0.97-1.06)	NS
CASI	31.8 (24.7)	44.7 (28.0)	-6.01	<0.001	1.02 (1.00-1.03)	0.012
NPI	17.4 (14.8)	8.0 (9.4)	7.66	<0.001	1.06 (1.04-1.07)	<0.001
VaMCI/PrVaD/PoVaD	3/88/64	187/561/378	23.43	<0.001	NA	
CVD subtypes	Mi 19/St 28/Sc 26/Bw 40/Cc 25/He 3/Ot 14	Mi 129/St 169/Sc 279/Bw 226/Cc 208/He 56/Ot 59	15.80	NS	NA	

VCI: vascular cognitive impairment; VH: visual hallucination; *N*: number of cases; NA: not applicable; NS: nonsignificance; OR: odds ratio. Adjusted ORs of variables were ORs adjusted for age and disease severity by Clinical Dementia Rating (CDR) scale; CDR-SB: sum of boxes of CDR; IADL: Instrumental Activities of Daily Living; MoCA: Montreal Cognitive Assessment; CASI: Cognitive Abilities Screening Instrument; NPI: total score of 12-domain Neuropsychiatric Inventory; VaMCI/PrVaD/PoVaD: vascular mild cognitive impairment/probable vascular dementia/possible vascular dementia; CVD: cerebrovascular disease; Mi: multi-infarct; St: strategic infarct; Sc: subcortical lacunes; Bw: Binswanger disease; Cc; complex combination; He: hemorrhage; Ot: others.

**Table 2:** Clinical manifestation of VCI patients with or without visual hallucinations (VHs).

	*N* (%)		Nonadjusted		Adjusted	
	VH+ (*N* = 155)	VH− (*N* = 1126)	*χ* ^2^	*p*	OR (95% CI)	*p*
DLB core features						
Fluctuation	90 (58.1)	309 (27.4)	59.57	<0.001	2.48 (1.69-3.63)	<0.001
Parkinsonism	75 (48.4)	371 (33.0)	14.25	<0.001	1.85 (1.30-2.63)	0.001
RBD	38 (24.5)	73 (6.5)	55.98	<0.001	4.56 (2.86-7.26)	<0.001
≧2 core features^∗^	129 (83.2)	166 (14.8)	360.11	<0.001	26.01 (16.23-41.67)	<0.001
Current medication						
Anti-Parkinson	14 (9.0)	45 (4.0)	7.86	0.005	2.35 (1.22-4.52)	0.010
Antipsychotics	9 (5.8)	41 (3.6)	1.70	NS	1.73 (0.80-3.75)	NS
Antidementia	14 (9.0)	69 (6.1)	1.90	NS	1.12 (0.60-2.29)	NS
Vascular risk factors						
Hypertension	83 (53.5)	548 (48.7)	1.30	NS	1.47 (1.03-2.09)	0.032
Diabetes	61 (39.4)	320 (28.4)	7.80	0.005	1.86 (1.29-2.69)	0.001
Hyperlipidemia	40 (25.8)	261 (23.2)	0.52	NS	1.38 (0.92-2.06)	NS
Heart disease	27 (17.4)	119 (10.6)	66.33	0.012	2.12 (1.32-3.43)	0.002

VCI: vascular cognitive impairment; VH: visual hallucination; *N*: number of cases; NA: not applicable; NS: nonsignificance; OR: odds ratio. Adjusted ORs of variables were ORs adjusted for age, gender, and disease severity by Clinical Dementia Rating (CDR) scale; CI: confidence interval; DLB: dementia with Lewy bodies; RBD: REM sleep behavior disorder; ^∗^core features including fluctuation, parkinsonism, RBD, and VH; heart disease including coronary artery disease, heart failure, arrhythmia, and valvular heart disease.

**Table 3:** Cerebral CT/MRI manifestation of VCI patients with or without visual hallucinations (VHs).

	Mean (SD)		Nonadjusted		Adjusted	
	VH+ (*N* = 155)	VH− (*N* = 1126)	*t*/*χ*^2^	*p*	OR (95% CI)	*p*
Fazekas scale	2.2 (1.0)	1.9 (1.0)	2.91	0.004	1.07 (0.88-1.31)	NS
MTA scale	2.6 (1.1)	2.1 (1.2)	4.96	<0.001	1.27 (1.06-1.52)	0.010
Global atrophy scale	1.6 (0.8)	1.5 (0.7)	2.09	0.031	0.99 (0.75-1.30)	NS
Cerebral microbleeds^∗^, *N* (%)	29 (30.3)	305 (40.1)	4.95	0.026	0.63 (0.39-1.02)	NS
Evans' index	0.29 (0.04)	0.28 (0.05)	1.69	0.091	0.07 (0.00-14.50)	NS

VCI: vascular cognitive impairment; VH: visual hallucination; *N*: number of cases; NA: not applicable; NS: nonsignificance; MTA: medial temporal lobe atrophy; OR: odds ratio. Adjusted ORs of variables were ORs adjusted for age, gender, and disease severity by Clinical Dementia Rating scale. ^∗^Cerebral microbleeds in MRI T2 gradient imaging among 94 VH+ and 760 VH−.

## Data Availability

The data that support the findings of this study are available from the corresponding author upon reasonable request.
